# Biologically Effective Dose-Optimized Multi-Intensity-Modulated Proton Therapy: A Biologically Comparable Alternative to Proton Arc Therapy

**DOI:** 10.1016/j.ijpt.2026.101321

**Published:** 2026-05-08

**Authors:** Nimita Shinde, Yanan Zhu, Wei Wang, Wangyao Li, Yuting Lin, Gregory N. Gan, Christopher Lominska, Ronny Rotondo, Ronald C. Chen, Hao Gao

**Affiliations:** 1 Department of Radiation Oncology, University of Texas Southwestern Medical Center, Dallas, TX, USA; 2 School of Mathematics, Harbin Institute of Technology, Harbin, China; 3 Department of Radiation Oncology, University of Kansas Medical Center, Kansas City, KS, USA

**Keywords:** Biologically effective dose (BED), Proton arc therapy, IMPT, Treatment planning, Alternating direction method of multipliers (ADMM)

## Abstract

**Purpose:**

Proton spot-scanning arc therapy (ARC) is an emerging technique that can enhance high-dose conformity to targets compared with standard intensity-modulated proton therapy (IMPT). Although proton ARC is highly desirable, it is not yet clinically available. Multiple IMPT plans delivered over different fractions and optimized simultaneously provide a practical means to approximate ARC-plan quality. However, existing multiple IMPT approaches are not optimized to be biologically comparable to proton ARC because they neglect the fractionation effect during treatment planning. This work proposes a biologically optimized multiple IMPT (multi-IMPT) framework that achieves comparable performance to proton ARC in terms of the biologically effective dose (BED). This is achieved through direct optimization of BED by explicitly incorporating the fractionation effect during planning, thereby ensuring biological comparability between multi-IMPT and proton ARC treatments.

**Materials and Methods:**

The proposed multi-IMPT method utilizes a different subset of limited number of beam angles in each fraction for dose delivery. Due to the different dose delivered to organs at risk (OAR) in each fraction, the BED delivered to OAR and the physical dose delivered to target is optimized in each fraction. The BED-based multi-IMPT inverse optimization problem is solved via the iterative convex relaxation method and the alternating direction method of multipliers. The effectiveness of the proposed multi-IMPT method is evaluated in terms of BED objectives in comparison with ARC and IMPT.

**Results:**

Multi-IMPT provided similar plan quality with ARC. For example, multi-IMPT provided better OAR sparing and slightly better target dose coverage for the prostate case; similar dose distribution for the lung case; slightly worse dose coverage for the brain case; better dose coverage but slightly higher BED in OAR for the head-and-neck case.

**Conclusion:**

A multi-IMPT approach is proposed that delivers ARC-comparable plan quality under the evaluated conditions in terms of BED.

## Introduction

Passive-scattering-based proton arc radiotherapy was first proposed in^1^ demonstrating its potential to improve target dose conformity over intensity-modulated proton therapy (IMPT). However, the implementation of passive-scattering-based proton arc radiotherapy^2^, ^3^, ^4^ faced several technical challenges, including the need for continuous adjustments of the beam compensator and range modulation wheel during gantry rotation.

To overcome these challenges, proton spot-scanning arc therapy (ARC) was developed.^5^, ^6^, ^7^, ^8^, ^9^, ^10^ ARC utilizes modern scanning nozzles and eliminates the need for a beam compensator and range modulation wheel. This advancement enables highly conformal dose distributions and helps to spare organs-at-risk (OAR) adjacent to the target.^5^, ^6^, ^7^, ^8^, ^11^, ^12^, ^13^ However, ARC delivery often involves frequent energy changes during gantry rotation, which can reduce treatment efficiency due to energy-switching delays.

Despite its dosimetric advantages, proton ARC is not yet clinically available at most proton therapy centers due to delivery complexity, energy layer switching requirements, and limited vendor support. This gap has motivated the development of alternative strategies that can approximate the angular diversity of ARC using existing spot-scanning proton systems. One such alternative method to proton ARC was recently introduced in,^14^ where IMPT plan is used to deliver a treatment comparable to proton ARC by utilizing different beam angles across different fractions and optimizing the physical dose delivered. Their study showed that the proton ARC and their proposed IMPT plan were comparable in terms of target robustness and normal tissue complication probability. The work^14^ also analyzed the biologically effective dose (BED) using the linear-quadratic (LQ) model. Calculating BED is particularly relevant for treatment strategies involving multiple fractions, as BED more accurately captures the effects of fractionation. However, their method optimized the physical dose to OAR and calculated BED only a posteriori.

In fractionated radiotherapy, biological response depends not only on cumulative physical dose but also on the temporal distribution of dose across fractions. This effect is particularly pronounced for OAR with low α/β ratios, where higher per-fraction dose leads to disproportionately larger biological damage under the LQ model. Fraction-specific variation in dose delivery, that occurs when different beam angle subsets are used across fractions, can therefore result in biologically meaningful differences even when cumulative physical dose is similar. In such settings, optimization based solely on physical dose may fail to capture clinically relevant fractionation effects, motivating direct optimization of BED.

This study proposes a method called multi-IMPT, which is biologically comparable to ARC in terms of plan quality. Conceptually similar to the framework in,^14^ multi-IMPT alternates between multiple IMPT plans with distinct subsets of beam angles across treatment fractions. The key distinction is that this work directly optimizes the BED (explicitly incorporating fractionation effects using the LQ model) to ensure biologically similar plan to proton ARC. By focusing on biological dose optimization, the proposed method provides a rigorous framework for comparing the biological effectiveness of multi-fraction IMPT and proton ARC. In treatment sites such as prostate, head-and-neck, and centrally located lung tumors, critical organs surround the target in multiple directions, and normal tissue sparing relies on distributing the dose over many beam angles rather than avoiding a single direction. Standard IMPT typically employs a fixed set of beam angles across all fractions, limiting its ability to exploit temporal angular averaging and potentially leading to higher cumulative biological dose to adjacent OAR. While proton ARC achieves improved OAR sparing through angular averaging within each fraction, multi-IMPT seeks to approximate this effect by varying beam directions across fractions so that normal tissues receive dose from different angles over time. This strategy is most relevant for conventionally fractionated treatments, where normal tissue toxicity is driven by cumulative fractionation effects rather than by single-fraction dose limits.

## Methods

### Optimization framework overview and motivation

The goal of the proposed framework is to design a fractionated proton therapy plan that achieves target coverage while minimizing normal tissue toxicity, explicitly accounting for fractionation-dependent biological effects to achieve performance comparable to proton ARC. To achieve this, the proposed work directly optimizes the BED delivered to OAR while maintaining clinically acceptable target dose coverage. The proposed multi-IMPT strategy delivers treatment using multiple fixed-beam IMPT plans, each corresponding to a different subset of equidistant beam angles. These plans are alternated across fractions so that normal tissues receive dose from different directions over time. From an optimization perspective, the problem consists of determining the spot intensities for each subplan such that the cumulative BED to OAR is minimized or constrained, while ensuring that each fraction delivers an appropriate physical dose to the target. The resulting formulation allows the optimization to be decomposed across fractions, while still enforcing cumulative biological objectives.

### Defining parameters and decision variables in the proposed multi‐IMPT optimization problem

All quantities associated with the proposed optimization problem are defined below.

*Parameters and decision variable:*

1. $M=\left\{1,\ldots ,M\right\}$: set of indices of OAR.
2. For $m\in M,n_{m}$: number of voxels in m-th OAR.
3. $A^{\mathrm{tm}}\in R^{n_{m}\times k_{t}}$: dose deposition matrix for m-th OAR during fraction t; $k_{t}$ is the number of beams in the active fields in fraction t; $A_{j}^{m}$: j-th row of the matrix $A^{m}$ and corresponds to the j-th voxel in OAR m.
4. $A^{t0}\in R^{n_{0}\times k_{t}}$: dose deposition matrix corresponding to the target during fraction t; $n_{0}$ is the number of voxels in the target volume.
5. T: number of fractions
6. (Decision variable) $u_{t}\in R^{k_{t}}$: spot intensity vector in fraction t, for t=1,…,T.

*BED*^15^, ^16^, ^17^ *and physical dose (d)*:

1. *BED delivered to OAR:* For OAR m, let $\alpha_{m},\beta_{m}$ be the parameters of the well-known LQ-model that is used to define BED. Define $\rho_{m}=1/\left(\alpha_{m}/\beta_{m}\right)$. Under the LQ model, the total BED delivered to the j-th voxel in OAR m is $\sum_{t=1}^{T}(A_{j}^{\mathrm{tm}}u_{t}+\rho_{m}{\left(A_{j}^{\mathrm{tm}}u_{t}\right)}^{2})$. During the experiments, $\alpha_{m}/\beta_{m}$ value is set to 2 Gy for all m.
2. *Physical dose delivered to target:* The physical dose delivered to each target voxel $j\in \left[n_{0}\right]$ in each fraction is calculated as $d_{j}^{t0}=A_{j}^{t0}u_{t}$.

### Optimization problem

The proposed multi-IMPT optimization problem is formulated as

(1) $$\begin{array}{c} \underset{u_{t}}{\min}\sum_{t=1}^{T}\mathrm{||}A^{t0}u_{t}-\mathrm{px}|{|}_{2}^{2}\;s.t.\sum_{t=1}^{T}A_{j}^{\mathrm{tm}}u_{t}\;+\sum_{t=1}^{T}\rho_{m}{\left(A_{j}^{\mathrm{tm}}u_{t}\right)}^{2}\leq \mathrm{BE}D_{\max}^{m}\forall m\in M_{1},j\in \left[n_{m}\right],\sum_{j=1}^{n_{m}}\sum_{t=1}^{T}A_{j}^{\mathrm{tm}}u_{t}\;+\sum_{j=1}^{n_{m}}\sum_{t=1}^{T}\rho_{m}{\left(A_{j}^{\mathrm{tm}}u_{t}\right)}^{2}\leq n_{m}\times \mathrm{BE}D_{\mathrm{mean}}^{m}\forall m\in M_{2},\#\sum_{t=1}^{T}A_{j}^{\mathrm{tm}}u_{t}\;+\sum_{t=1}^{T}\rho_{m}{\left(A_{j}^{\mathrm{tm}}u_{t}\right)}^{2}\leq \mathrm{BE}D_{\mathrm{dv}}^{m}\forall m\in M_{3},j\in \Omega^{m},A_{j}^{t0}u_{t}\;\geq d_{\mathrm{dv}}^{0}\forall j\in \Omega^{0},t\in \left[T\right],A_{j}^{t0}u_{t}\;\leq 1.1\mathrm{px}\forall j\in \left[n_{0}\right],t\in \left[T\right],u_{t}\in \left\{0\right\}\cup \left[g,+\infty \right\}\forall t=1,\ldots ,T. \end{array}$$

The multi-IMPT model (Equation (1)) is non-convex with quadratic constraints. The objective function forms a DVH-based objective and minimizes the least squares error between prescribed dose and actual dose delivered to the target. The first 3 constraints describe clinical constraints on BED delivered to the OAR. The next 2 constraints provide DVH-min and maximum dose constraint for the target. Finally, the last constraint in Equation (1) defines a minimum-monitor-unit constraint^18^, ^19^, ^20^, ^21^, ^22^, ^23^ for $u_{t}$ with g as the minimum-monitor-unit threshold to ensure plan deliverability. The first 5 constraints are described in detail below:

1. *BED-max constraint for OAR*^24^, ^25^, ^26^, ^27^*:* Let $M_{1}$be the set of OAR that are highly sensitive to radiation, and their function is hampered even when a single voxel is damaged by radiation. For such OAR, the BED-max constraint bounds the maximum BED ($\mathrm{BE}D_{\max}^{m}$) delivered to each voxel in OAR m. Thus, BED-max constraint is defined as
  $$\sum_{t=1}^{T}A_{j}^{\mathrm{tm}}u_{t}+\sum_{t=1}^{T}\rho_{m}{\left(A_{j}^{\mathrm{tm}}u_{t}\right)}^{2}\leq \mathrm{BE}D_{\max}^{m}\forall m\in M_{1},j\in \left[n_{m}\right].$$
2. *BED-mean constraint for OAR*^24^, ^25^, ^26^, ^27^*:* Let $M_{2}$ be the set of OAR whose small portion can be damaged without affecting their function. For such OAR, the BED-mean constraint bounds the mean BED ($\mathrm{BE}D_{\mathrm{mean}}^{m}$) delivered to all voxels in OAR m. The BED-mean constraint is defined as
  $$\sum_{j=1}^{n_{m}}\sum_{t=1}^{T}A_{j}^{\mathrm{tm}}u_{t}+\sum_{j=1}^{n_{m}}\sum_{t=1}^{T}\rho_{m}{\left(A_{j}^{\mathrm{tm}}u_{t}\right)}^{2}\leq n_{m}\times \mathrm{BE}D_{\mathrm{mean}}^{m}\forall m\in M_{2}.$$
3. *BED-DVH max constraint for OAR*^24^, ^25^, ^26^, ^27^: Consider a set $M_{3}\;$of OAR. The BED-DVH constraints states that for any OAR $m\in M_{3}$, at most p fraction of voxels should receive BED larger than $\mathrm{BE}D_{\mathrm{dv}}^{m}$, ie, $\mathrm{BE}D_{j}^{m}=\sum_{t=1}^{T}A_{j}^{\mathrm{tm}}u_{t}+\sum_{t=1}^{T}\rho_{m}{\left(A_{j}^{\mathrm{tm}}u_{t}\right)}^{2}\geq \mathrm{BE}D_{\mathrm{dv}}^{m}$ for at most $p\times n_{m}$ voxels. One of the commonly used techniques to define the BED-DVH max constraint is to first define the set of indices (called active index set) of voxels that violate the constraint. More precisely, let $\left[n_{m}^{\prime }\right]$ be the set of indices of voxels that are sorted in descending order of the BED delivered to the voxels in OAR m. The active index set is then defined as
  $$\Omega^{m}=\{j\in \left[n_{m}^{\prime }\right]|j\geq p\times n_{m},\mathrm{BE}D_{j}^{m}\geq \mathrm{BE}D_{\mathrm{dv}}^{m}\}.$$
  If the active index set, $\Omega^{m}$, is non-empty, then BED-DVH max constraint is defined as
  $$\sum_{t=1}^{T}A_{j}^{\mathrm{tm}}u_{t}+\sum_{t=1}^{T}\rho_{m}{\left(A_{j}^{\mathrm{tm}}u_{t}\right)}^{2}\leq \mathrm{BE}D_{\mathrm{dv}}^{m}\forall m\in M_{3},j\in \Omega^{m}.$$
4. *DVH min constraint for target*^28^, ^29^: DVH min constraint ensures that at least p fraction of the target voxels receive physical dose larger than $d_{\mathrm{dv}}^{0}$ in each fraction t, ie, $d_{j}^{t0}\geq d_{\mathrm{dv}}^{0}$, for at least $p\times n_{0}$ voxels. To define the DVH min constraint, the active index set for the target is defined as
  $$\Omega^{0}=\{j\in \left[n_{0}^{\prime }\right]|j\leq p\times n_{m},d_{j}^{t0}\leq d_{\mathrm{dv}}^{0}\},$$
  where $\left[n_{0}^{\prime }\right]$ is the set of indices of the target voxels sorted in the descending order of the dose delivered. The DVH min constraint is then defined as
  $$d_{j}^{t0}\geq d_{\mathrm{dv}}^{0}\forall j\in \Omega^{0}.$$
5. *Max dose for target:* Finally, max dose constraint bounds the maximum physical dose that can be tolerated by target dose in each fraction. In the proposed model, this constraint is defined as

$$d_{j}^{t0}=A_{j}^{t0}u_{t}\leq 1.1\mathrm{px}\forall j\in \left[n_{0}\right],t\in \left[T\right],$$

ie, the dose delivered to each target voxel j in each fraction t should not exceed 1.1 times the prescribed physical dose (px).

Before presenting the solution methodology, a comparison of Equation (1) with ARC is first provided.

*Comparison with ARC:* In the multi-IMPT formulation (Equation (1)), the decision variable (spot intensity vector $u_{t}$) varies across fractions because each fraction uses a different, smaller subset of beam angles. This contrasts with the ARC approach, where all beam angles are active in every fraction, resulting in same dose delivery u per fraction. Additionally, Equation (1) can be reformulated to be separable in t (as discussed in Section 2.5), allowing the problem to be divided into T independent, smaller optimization problems, each with its own decision variable $u_{t}$. In the ARC model, however, the decision variable u includes spot intensities from all beam angles simultaneously, creating a much larger, single optimization problem. Consequently, each $u_{t}$ in Equation (1) is far smaller in dimension than u in the ARC formulation, making the multi-IMPT subproblems computationally cheaper to solve.

### Beam angle subset selection, fractionation, and plan cycling in multi‐IMPT

In the proposed multi-IMPT framework, each treatment fraction is delivered using a fixed subset of 4 proton beam angles. The use of 4 beams per fraction represents a deliberate compromise between angular diversity and per-fraction delivery complexity and is consistent with standard IMPT practice, where treatment plans typically employ 3 to 5 beams per fraction. This choice limits per-fraction delivery time and plan complexity while still allowing sufficient angular variation across fractions to achieve effective normal tissue sparing.

A total of 6 beam-angle subsets are constructed using interlaced angular offsets of 15°, such that the union of all subsets uniformly samples the full 360° angular space. These 6 IMPT plans are delivered sequentially and cyclically across the full course of treatment. For example, fraction 1 uses plan 1, fraction 2 uses plan 2, and so on, with the sequence repeated until all fractions are delivered. As a result, each beam-angle subset contributes equally to the cumulative physical dose and BED. This delivery strategy distributes angular sampling temporally across fractions, providing an analog to the angular averaging achieved by proton ARC within each fraction, while preserving the fixed-beam, clinically deliverable nature of IMPT.

Although the optimization variables are defined on a per-fraction basis and the problem can be decomposed computationally across beam-angle subsets, fractions remain biologically coupled through the cumulative BED objective and constraints. The BED formulation explicitly sums dose contributions from all fractions for each voxel, ensuring that dose delivered in 1 fraction influences the allowable dose in subsequent fractions. Thus, separability is a computational feature of the solution strategy rather than an indication of biological independence across fractions. In addition, the quadratic term in the BED formulation penalizes large per-fraction doses, discouraging temporally concentrated OAR dose patterns.

### Solution algorithm

To solve Equation (1), the following auxiliary variables are introduced: $z_{j}^{\mathrm{tm}}=A_{j}^{\mathrm{tm}}u_{t}$ for all $j\in \left[n_{m}\right],m\in M_{1}\cup M_{2}\cup M_{3},t\in \left[T\right]$, and $z_{j}^{t0}=A_{j}^{t0}u_{t}$ for all $j\in \left[n_{0}\right],t\in \left[T\right]$. Equation (1) is then re-written as

(2) $$\begin{array}{c} \underset{u_{t},z_{j}^{\mathrm{tm}},z_{j}^{t0}\;}{\min}\sum_{t=1}^{T}\mathrm{||}A^{t0}u_{t}-\mathrm{px}|{|}_{2}^{2}s.t.\sum_{t=1}^{T}\left(z_{j}^{\mathrm{tm}}\right)\;+\sum_{t=1}^{T}\rho_{m}{\left(z_{j}^{\mathrm{tm}}\right)}^{2}\leq \mathrm{BE}D_{\max}^{m}\forall m\in M_{1},j\in \left[n_{m}\right],\;\sum_{j=1}^{n_{m}}\sum_{t=1}^{T}\left(z_{j}^{\mathrm{tm}}\right)\;+\sum_{j=1}^{n_{m}}\sum_{t=1}^{T}\rho_{m}{\left(z_{j}^{\mathrm{tm}}\right)}^{2}\leq n_{m}\times \mathrm{BE}D_{\mathrm{mean}}^{m}\forall m\in M_{2},\sum_{t=1}^{T}\left(z_{j}^{\mathrm{tm}}\right)\;+\sum_{t=1}^{T}\rho_{m}{\left(z_{j}^{\mathrm{tm}}\right)}^{2}\leq \mathrm{BE}D_{\mathrm{dv}}^{m}\forall m\in M_{3},j\in \Omega^{m},\#z_{j}^{t0}\;\geq d_{\mathrm{dv}}^{0}\forall j\in \Omega^{0},t\in \left[T\right],z_{j}^{t0}\leq 1.1\mathrm{px}\forall j\in \left[n_{0}\right],t\in \left[T\right],z_{j}^{\mathrm{tm}}\;=A_{j}^{\mathrm{tm}}u_{t}\forall j\in \left[n_{m}\right],m\in M_{1}\cup M_{2}\cup M_{3},t\in \left[T\right],z_{j}^{t0}\;=A_{j}^{t0}u_{t}\forall j\in \left[n_{0}\right],t\in \left[T\right],\;u_{t}\in \left\{0\right\}\cup \left[g,+\infty \right\}\forall t=1,\ldots ,T. \end{array}$$

Equation (2) can now be solved via the iterative convex relaxation method^30^, ^31^ and alternating direction method of multipliers method.^32^, ^33^ The method involves iteratively updating the active index sets (as defined in Section 2.3) followed by sequentially updating each decision variable in the problem. To do so, the augmented Lagrangian of Equation (2) is defined as

(3) $$\begin{array}{c} \underset{\;}{\min}\frac{w_{0}}{n_{0}}\sum_{t=1}^{T}\mathrm{||}A^{t0}u_{t}-\mathrm{px}|{|}_{2}^{2}\;+\frac{\mu_{1}}{2}\sum_{t=1}^{T}\sum_{m\in M_{1}\cup M_{2}\cup M_{3}}\frac{w_{m}}{n_{m}}\mathrm{||}A^{\mathrm{tm}}u_{t}-z^{\mathrm{tm}}\;+\lambda^{\mathrm{tm}}|{|}_{2}^{2}+\frac{\mu_{2}}{2}\sum_{t=1}^{T}\frac{w_{0}^{1}}{n_{0}}\mathrm{||}A^{t0}u_{t}-z^{t0}+\lambda^{t0}|{|}_{2}^{2}+\frac{\mu_{3}}{2}\sum_{t=1}^{T}\frac{w_{0}^{2}}{n_{0}}\mathrm{||}A^{t0}u_{t}\;-1.1\mathrm{px}+\gamma^{t}|{|}_{2}^{2}+\frac{\mu_{4}}{2}\sum_{t=1}^{T}\mathrm{||}u_{t}-y_{t}+\zeta_{t}|{|}_{2}^{2}s.t.\sum_{t=1}^{T}\left(z_{j}^{\mathrm{tm}}\right)\;+\sum_{t=1}^{T}\rho_{m}{\left(z_{j}^{\mathrm{tm}}\right)}^{2}\leq \mathrm{BE}D_{\max}^{m}\forall m\in M_{1},j\in \left[n_{m}\right],\sum_{j=1}^{n_{m}}\sum_{t=1}^{T}\left(z_{j}^{\mathrm{tm}}\right)\;+\sum_{j=1}^{n_{m}}\sum_{t=1}^{T}\rho_{m}{\left(z_{j}^{\mathrm{tm}}\right)}^{2}\leq n_{m}\times \mathrm{BE}D_{\mathrm{mean}}^{m}\forall m\in M_{2},\#\sum_{t=1}^{T}\left(z_{j}^{\mathrm{tm}}\right)\;+\sum_{t=1}^{T}\rho_{m}{\left(z_{j}^{\mathrm{tm}}\right)}^{2}\leq \mathrm{BE}D_{\mathrm{dv}}^{m}\forall m\in M_{3},j\in \Omega^{m},z_{j}^{t0}\;\geq d_{\mathrm{dv}}^{0}\forall j\in \Omega^{0},t\in \left[T\right],y_{t}\in \left\{0\right\}\cup \left[g,+\infty \right\}\forall t=1,\ldots ,T. \end{array}$$

In Equation (3), $u_{t}$, $z^{\mathrm{tm}}$, $z^{t0}$, $y_{t}$ are primal variables and $\lambda^{\mathrm{tm}}$, $\lambda^{t0}$, $\gamma^{t}$, $\zeta_{t}$ are dual variables. Algorithm 1 provides a brief outline of the optimization method that solves Equation (3). Each step is explained in detail in Appendix A.

Algorithm 1 Optimization method for solving Equation (3)

**Table:**

### Materials

The comparison of multi-IMPT with ARC in terms of the BED was demonstrated for 4 clinical cases. For ARC, the beam angles were spaced at 15° intervals over a 360° rotation. For multi-IMPT, 6 beam angle combinations were used: (0°, 90°, 180°, 270°), (15°, 105°, 195°, 285°), (30°, 120°, 210°, 300°), (45°, 135°, 225°, 315°), (60°, 150°, 240°, 330°), and (75°, 165°, 255°, 345°), generating 6 unique IMPT plans. The selection of 6 IMPT plans represents a balance between angular sampling density and computational feasibility, providing sufficient angular coverage (every 15° when interlaced) to emulate the proton ARC delivery. Each of these plans were delivered sequentially and cyclically for the same total number of fractions as the ARC plan. For example, in the lung case, both ARC and multi-IMPT consist of 30 fractions, with the 6 multi-IMPT plans delivered sequentially and repeated 5 times over the full course of treatment.

For comparison, a standard IMPT plan was also generated for each case using a fixed 4-beam configuration corresponding to the angles (0°, 90°, 180°, 270°) for the lung and prostate case and angles (45°, 135°, 225°, 315°) for the brain and HN case. This baseline IMPT plan was optimized using the same objective function, constraint definitions, BED parameters, and minimum monitor unit constraints as used in the multi-IMPT formulation, but without fraction-varying beam angles. This ensures that differences between IMPT and multi-IMPT arise solely from temporal angular variation rather than from differing optimization settings.

All ARC, multi-IMPT, and IMPT plans were generated by solving Equation (1) using the method described in Algorithm 1. Proton dose calculation was performed using matRad’s^34^ analytical pencil beam algorithm with the default generic clinical proton beam model. Depth-dose curves, range-energy relationships, and lateral beam spread parameters were taken directly from the matRad beam library without modification. No range shifters, apertures, or additional beamline customizations were used. Spot placement and energy layer discretization followed matRad’s default settings. All dose calculations were performed on a 3 mm^3^ grid.

**Figure 1:**
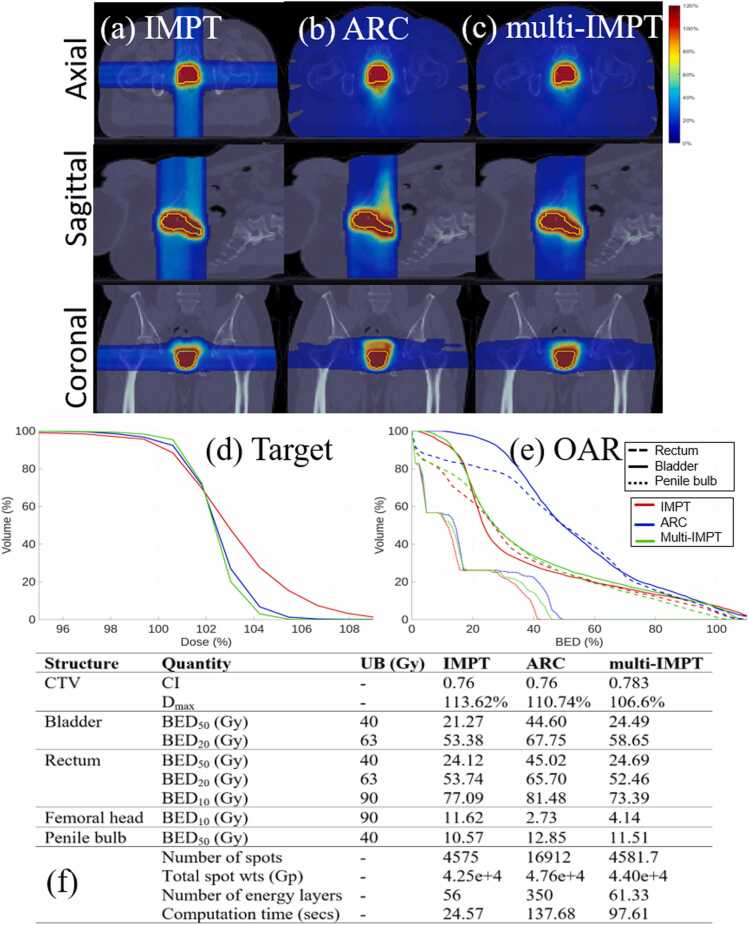
*Prostate.* (a)-(c) Dose plots for IMPT, ARC, and multi-IMPT methods, respectively. (d) DVH plot for the target. (e) BED-DVH plot for OAR. (f) Comparison of plan quality for IMPT, ARC, and the proposed multi-IMPT method. UB indicates upper bound of the BED (in Gy). The deliverability metrics (number of spots, total spots weights, and number of energy layers) are reported on a per-fraction basis. For multi-IMPT, values represent the average across all fractions.

**Figure 2:**
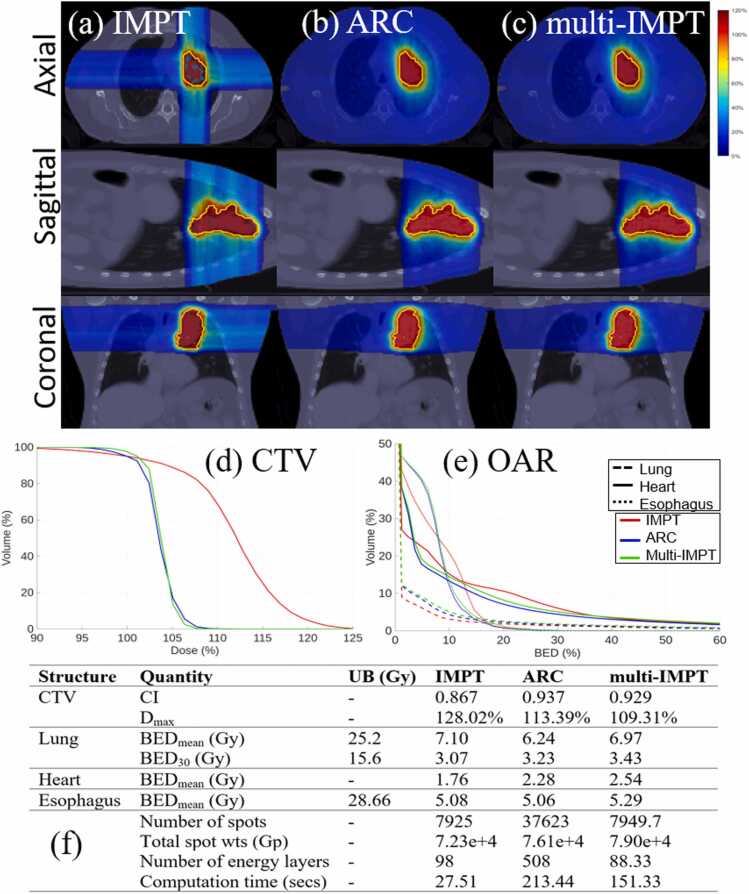
*Lung.* (a)-(c) Dose plots for IMPT, ARC, and multi-IMPT methods, respectively. (d) DVH plot for the target. (e) BED-DVH plot for OAR. (f) Comparison of plan quality for IMPT, ARC, and the proposed multi-IMPT method. UB indicates upper bound of the BED (in Gy). The deliverability metrics (number of spots, total spots weights, and number of energy layers) are reported on a per-fraction basis. For multi-IMPT, values represent the average across all fractions.

**Figure 3:**
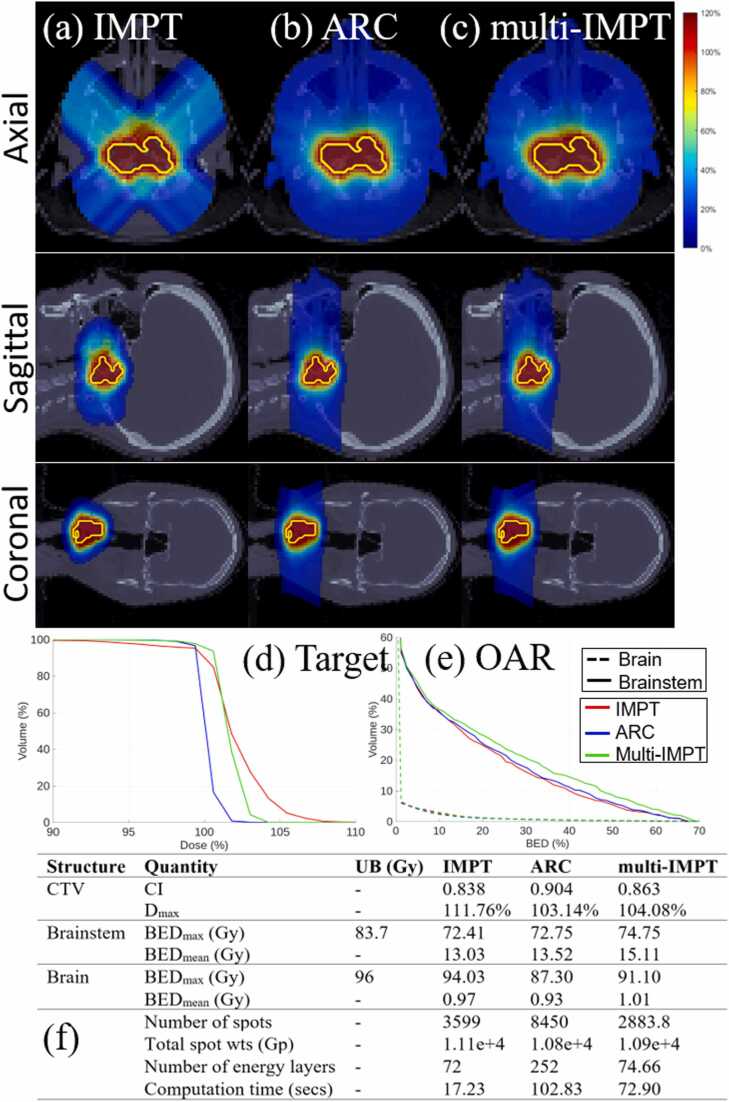
*Brain.* (a)-(c) Dose plots for IMPT, ARC, and multi-IMPT methods, respectively. (d) DVH plot for the target. (e) BED-DVH plot for OAR. (f) Comparison of plan quality for IMPT, ARC, and the proposed multi-IMPT method. UB indicates upper bound of the BED (in Gy). The deliverability metrics (number of spots, total spots weights, and number of energy layers) are reported on a per-fraction basis. For multi-IMPT, values represent the average across all fractions.

**Figure 4:**
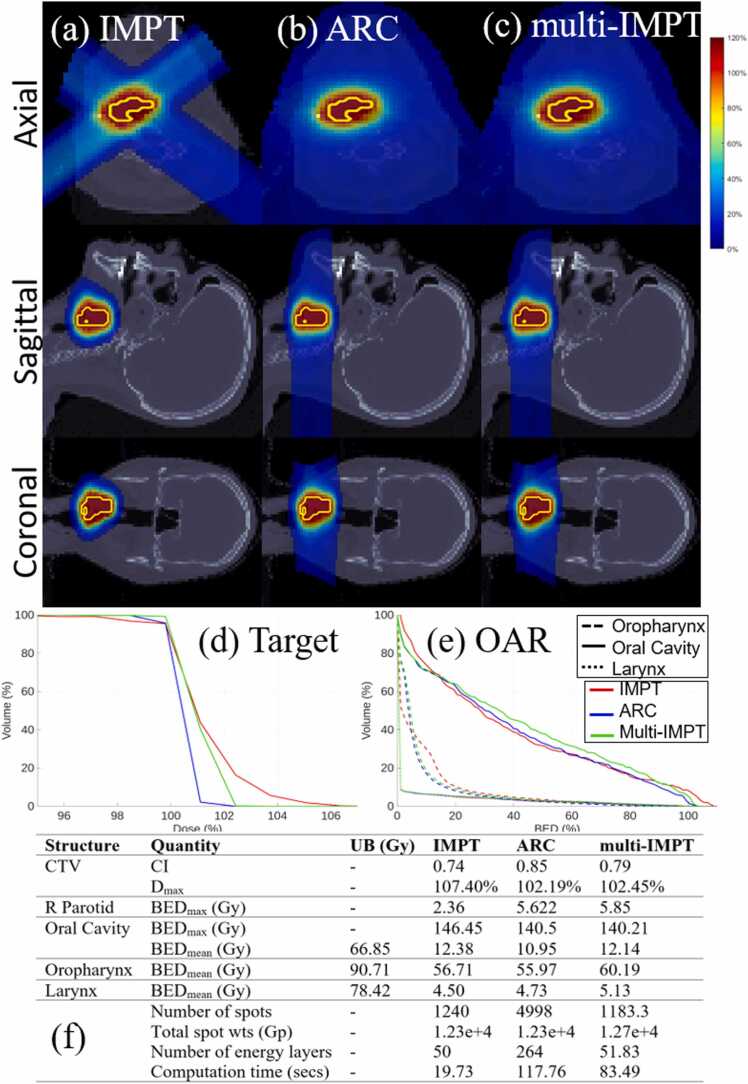
*HN.* (a)-(c) Dose plots for IMPT, ARC, and multi-IMPT methods, respectively. (d) DVH plot for the target. (e) BED-DVH plot for OAR. (f) Comparison of plan quality for IMPT, ARC, and the proposed multi-IMPT method. UB indicates upper bound of the BED (in Gy). The deliverability metrics (number of spots, total spots weights, and number of energy layers) are reported on a per-fraction basis. For multi-IMPT, values represent the average across all fractions.

The prescribed dose and number of fractions for the 4 clinical cases were: (1) prostate case (1.8 Gy x 25 fractions), (2) lung case (2 Gy x 30 fractions), (3) brain case (1.2 Gy x 60 fractions), (4) head-and-neck (HN) case (2 Gy x 35 fractions). The upper bounds (right-hand side) of each constraint in Equation (1) are stated in Figure 1, Figure 2, Figure 3, Figure 4 for the respective test cases. BED_p_ denotes that at most *p*% of OAR voxels should receive BED greater than the value defined as the upper bound. For a fair comparison, all plans were normalized so that at least 95% of the target volume receives 100% of the prescribed dose, and all reported metrics correspond to the post-normalization plans. To quantify the plan quality, the following quantities were compared: (a) conformity index (CI), (b) maximum dose delivered to tumor (D_max_), (c) mean and max BED delivered to OAR. CI was defined as $V_{100}^{2}/\left(V\times {V\prime }_{100}\right)$, where $V_{100}$ is the target volume that receives at least 100% of the prescribed dose, V is the target volume, and ${V\prime }_{100}$ is the total volume that receives at least 100% of the prescribed dose. Deliverability metrics were additionally reported, including the number of spots, number of energy layers, and total spot weights. Total spot weights are expressed in giga-protons (Gp) and represent the sum of all optimized spot intensities across all beamlets and beam angles. All deliverability metrics are reported on a per-fraction basis. For multi-IMPT, these values correspond to the average per fraction across all 6 interlaced plans.

To evaluate robustness to setup and range uncertainties, an uncertainty analysis was performed for all clinical cases and all 3 planning approaches (IMPT, ARC, and multi-IMPT). Setup perturbations of ±3 mm were applied independently along each of the 3 orthogonal directions (left-right, anterior-posterior, and superior-inferior), and a ±3.5% range uncertainty was applied to account for proton stopping power estimation errors. These perturbations were evaluated as independent scenarios, resulting in 9 uncertainty scenarios per plan. For each scenario, dose distributions were recalculated without re-optimization. BED metrics were then computed using the perturbed dose distributions.

To evaluate potential temporal dose heterogeneity, per-fraction physical dose metrics were analyzed. For IMPT and ARC, the delivered dose distribution is identical across fractions; therefore, per-fraction values correspond to the nominal plan. For multi-IMPT, since 6 distinct subplans are delivered cyclically, per-fraction statistics were computed across these 6 subplans. For OAR and target metrics (D_max_, D_mean_, and D_p_), the maximum single-fraction value and the mean ± standard deviation across subplans were calculated. This analysis was performed to assess potential single-fraction dose concentration in multi-IMPT that may influence BED. This analysis is based on the 6 predefined beam-angle subsets delivered in a cyclic sequence, which represents an idealized and periodic delivery pattern (see Section 2.4 for details). In clinical practice, fraction-to-fraction variations in patient anatomy and setup may introduce deviations from this regular pattern, potentially altering the temporal dose distribution. Therefore, the presented per-fraction analysis (Section 3.4) should be interpreted as a controlled evaluation under structured delivery conditions rather than a comprehensive characterization of all possible temporal variations.

## Results

### Comparison of multi‐IMPT against ARC

Across all 4 clinical cases, multi-IMPT achieved plan quality comparable to ARC in terms of both target coverage and BED-based OAR sparing, with case-dependent differences in conformity and high-dose control. For target coverage, ARC demonstrated the highest conformity in the brain and HN cases, whereas multi-IMPT provided similar or slightly improved conformity in the prostate case and comparable conformity in the lung case. In the prostate case (Figure 1), CI was slightly higher with multi-IMPT (0.783 vs 0.76), while in the brain case (Figure 3), ARC achieved superior conformity (0.904 vs 0.863). In the HN case (Figure 4), ARC also showed better CI (0.85 vs 0.79). Across cases, differences in maximum target dose (Dmax) were modest; multi-IMPT reduced Dmax relative to ARC in the prostate and lung cases, while values were nearly identical in HN and slightly higher in the brain case. DVH plots confirm these trends, with multi-IMPT closely tracking ARC for target coverage but showing slightly reduced conformity in geometrically complex cases such as the brain.

With respect to OAR sparing, the relative performance was case-dependent. In the prostate case (Figure 1), multi-IMPT achieved substantially lower BED metrics for bladder and rectum compared to ARC, with pronounced reductions in intermediate- and high-dose exposure. In the lung case (Figure 2), ARC demonstrated slightly lower mean BED for lung and heart, although differences remained small and within clinically enforced upper bounds. In the brain case (Figure 3), ARC provided modestly improved BED control for the brain and brainstem. In the HN case (Figure 4), OAR BED metrics were broadly comparable between ARC and multi-IMPT, with differences generally within a few Gy and without clinically meaningful deviation from constraint limits. DVH plots corroborate the tabulated data, showing similar dose distributions with small variations in conformity and OAR high-dose regions.

Importantly, deliverability metrics differ substantially between the 2 techniques. ARC consistently required significantly higher per-fraction spot counts and energy layers (eg, lung: 37,623 spots and 508 energy layers for ARC vs 7949.7 spots and 88.33 energy layers for multi-IMPT). Thus, while cumulative angular sampling is equivalent over the treatment course, ARC exhibits substantially greater per-fraction delivery plan complexity. Finally, the computation time for multi-IMPT is also smaller than ARC since the multi-IMPT optimization problem can be divided into independent smaller subproblems (6 subproblems in this work). Overall, multi-IMPT achieves ARC-comparable biological plan quality while maintaining markedly lower per-fraction delivery burden.

### Comparison of multi‐IMPT against standard IMPT

Compared to standard IMPT, multi-IMPT consistently improved target conformity and reduced maximum dose across most clinical scenarios while maintaining comparable OAR BED metrics, as demonstrated in Figure 1, Figure 2, Figure 3, Figure 4. Across the 4 cases, CI values were generally higher with multi-IMPT than with IMPT (eg, lung: 0.929 vs 0.867; brain: 0.863 vs 0.838; HN: 0.79 vs 0.74), indicating improved dose conformity resulting from fraction-varying angular sampling. In the prostate case, conformity was similar but slightly improved with multi-IMPT. Maximum target dose was consistently lower with multi-IMPT across all cases (eg, lung: 109.31% vs 128.02%; brain: 104.08% vs 111.76%; HN: 102.45% vs 107.40%), reflecting improved high-dose control. Dose plots show reduced dose spillage into adjacent normal tissue with multi-IMPT compared to IMPT, particularly in the brain case.

OAR sparing between IMPT and multi-IMPT was generally comparable, with modest case-dependent trade-offs. In the prostate case, multi-IMPT achieved similar or slightly improved rectal and bladder BED metrics. In the lung case, lung and esophagus BED were slightly higher for IMPT, while heart mean BED was slightly lower for IMPT. In the brain case, IMPT achieved marginally lower OAR BED metrics but at the expense of inferior target conformity. In the HN case, OAR BED metrics for IMPT and multi-IMPT were largely within the same range. DVH plots confirm that differences in OAR exposure between IMPT and multi-IMPT are generally modest and remain within clinically defined bounds.

From a deliverability perspective, multi-IMPT preserves IMPT-like per-fraction complexity. Spot counts and energy layers per fraction for multi-IMPT are comparable to IMPT and substantially lower than ARC across all cases. Computationally, multi-IMPT requires roughly 6 times longer than standard IMPT, which is expected since multi-IMPT consists of 6 distinct IMPT plans.

Overall, multi-IMPT combines improved target conformity and reduced Dmax relative to standard IMPT with comparable OAR sparing that remains within clinically acceptable limits while maintaining per-fraction deliverability characteristics similar to IMPT. This positions multi-IMPT as an intermediate strategy that enhances cumulative angular coverage without incurring full per-fraction complexity of ARC.

### Robustness analysis under setup and range uncertainties

**Figure 5:**
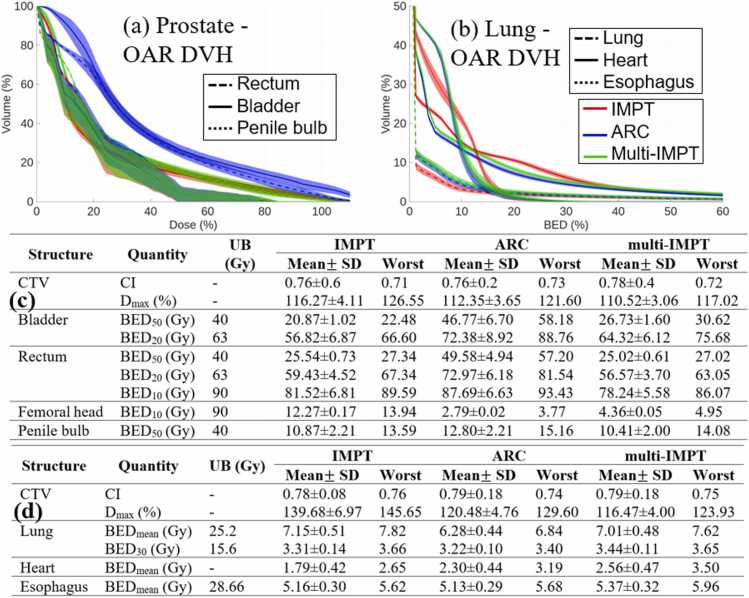
Uncertainty analysis for the prostate and lung cases. (a)-(b) OAR BED-DVH for prostate and lung, respectively. (c)-(d) Dosimetric comparison showing mean ± 1 standard deviation and worst-case values across uncertainty scenarios for prostate and lung, respectively.

Robustness evaluation under ±3 mm setup and ±3.5% range uncertainties shows that multi-IMPT maintains stability comparable to ARC across all 4 clinical cases. Representative robustness results for the prostate and lung cases are shown in Figure 5, while corresponding results for brain and HN cases are provided in Supplementary Figure S1. In the lung case, target conformity was similar among techniques (IMPT: 0.78 ± 0.08; ARC: 0.79 ± 0.18; multi-IMPT: 0.79 ± 0.18), while IMPT exhibited substantially higher Dmax variability (139.68% ± 6.97) compared to ARC (120.48% ± 4.76) and multi-IMPT (116.47% ± 4.00). The OAR BED metrics remained within tolerance limits for all methods, with modest variability. In the prostate case, multi-IMPT preserved target conformity comparable to ARC and maintained lower rectum and bladder BED than ARC. Dmax variability was lower for multi-IMPT (110.52% ± 3.06) than IMPT (116.27% ± 4.11). For brain and HN cases (Supplementary Figure S1), conformity and OAR BED variability were comparable among techniques, with no excessive sensitivity observed for multi-IMPT.

In addition to mean ± standard deviation across uncertainty scenarios, worst-case metrics were also evaluated. Worst-case values are defined as the most adverse outcomes observed across all uncertainty scenarios for each metric. The inclusion of worst-case evaluation shows that the relative performance trends between multi-IMPT and ARC remain consistent with those observed using mean values. For each metric, if 1 method demonstrates improved performance in terms of mean value, a similar trend is observed under worst-case conditions. Across all cases, BED-DVH plots under uncertainty show similar spread for ARC and multi-IMPT. Overall, multi-IMPT does not exhibit increased susceptibility to setup or range perturbations relative to ARC under the tested conditions.

### Per-fraction physical dose analysis

Because BED depends on per-fraction dose contributions, per-fraction physical dose metrics were evaluated to assess potential temporal dose concentration. IMPT and ARC deliver identical dose distributions each fraction; therefore, a single per-fraction value is reported. For multi-IMPT, statistics were computed across the 6 cyclically delivered subplans. Results for the prostate and lung cases are shown in Table 1, with brain and HN results provided in Supplementary Table S2.

**Table 1:** Per-fraction physical dose metrics for prostate and lung cases.

Case	Structure	Quantity	IMPT (per-fraction)	ARC (per-fraction)	Multi-IMPT (max across 6 subplans)	multi-IMPT (mean ± SD across 6 subplans)
Prostate	CTV	D_max_ (%)	113.62	110.73	113.62	109.10 ± 2.40
	Bladder	D_50_	0.60	1.08	0.72	0.67 ± 0.04
		D_20_	1.24	1.47	1.47	1.36 ± 0.10
	Rectum	D_50_	0.67	1.09	0.80	0.72 ± 0.06
		D_20_	1.24	1.44	1.25	1.23 ± 0.01
		D_10_	1.61	1.68	1.61	1.55 ± 0.03
	Fem head	D_10_	0.36	0.09	0.37	0.19 ± 0.15
	Penile bulb	D_50_	0.33	0.39	0.37	0.36 ± 0.01
Lung	CTV	D_max_ (%)	128.02	113.39	119.19	114.12 ± 2.97
	Lung	D_mean_	0.15	0.13	0.15	0.14 ± 0.01
		D_30_	0.09	0.09	0.03	0.01 ± 0.008
	Heart	D_mean_	0.03	0.05	0.06	0.05 ± 0.01
	Esophagus	D_mean_	0.13	0.13	0.15	0.13 ± 0.01

Since IMPT and ARC deliver an identical dose each fraction, a single per-fraction value is shown. Multi-IMPT statistics are computed across the 6 cyclically delivered subplans, with both the maximum single-fraction value and mean ± SD reported to quantify temporal dose variation. All OAR physical dose values are reported in Gy.

In the prostate case, multi-IMPT did not produce excessive single-fraction OAR doses. For example, rectum D_50_ was 0.72 ± 0.06 Gy (maximum 0.80 Gy) for multi-IMPT, compared to 1.09 Gy for ARC. Similar controlled variability was observed for bladder and other OAR. In the lung case, lung mean dose per fraction was 0.14 ± 0.01 Gy for multi-IMPT versus 0.13 Gy for ARC, with small inter-subplan variation. Target max dose variability for multi-IMPT remained moderate and comparable to ARC in both cases.

Consistent trends were observed in the brain and HN cases (Supplementary Table S2), where multi-IMPT exhibited modest inter-subplan variation without excessive single-fraction OAR dose variations. Across all 4 cases, maximum per-fraction OAR doses for multi-IMPT were comparable to ARC, indicating that inter-fraction angular redistribution does not introduce excessive temporal dose heterogeneity.

## Discussion

The multi-IMPT framework proposed in this work delivers treatment using multiple IMPT plans, each employing a distinct subset of beam angles. Across the full treatment course, these subsets collectively reproduce the same angular sampling as ARC, while enabling direct optimization of BED. The results demonstrate that multi-IMPT achieves dose coverage comparable to ARC in terms of both the BED delivered to OAR and the physical dose to the target across representative clinical cases.

Across the 4 clinical cases studied, performance was site-dependent. In the prostate case, multi-IMPT achieved substantially lower OAR BED compared to ARC while maintaining comparable or improved target conformity. In the lung and brain cases, multi-IMPT produced dose distributions similar to ARC, with small differences in conformity or OAR metrics that remained within clinically defined tolerance limits. In the HN case, ARC achieved slightly higher conformity, while OAR BED values were broadly comparable between methods. Overall, 3 out of the 4 cases showed multi-IMPT to be comparable or slightly superior to ARC, with the brain case being the exception where ARC performed marginally better. In this study, clinical significance is defined relative to established BED tolerance limits for OAR along with absolute numerical differences between competing plans. As a result, small variations in BED between ARC and multi-IMPT that remain well within these limits are not interpreted as clinically meaningful.

In the present formulation, OAR constraints are expressed in terms of BED, while the target objective is defined in terms of physical dose. This reflects a clinical practice in conventionally fractionated treatments, where tumor coverage is prescribed and evaluated in physical dose units. Under fixed α/β assumptions and conventional fractionation, tumor BED is a monotonic function of physical dose. Thus, optimizing physical dose ensures consistent BED ordering. In hypofractionated regimens or in settings with tumor α/β uncertainty, direct BED-based target optimization may warrant further investigation.

It is important to clarify that the demonstrated comparable BED between ARC and multi-IMPT pertains to cumulative BED across the entire treatment rather than to identical intra-fraction dose patterns. ARC inherently achieves angular averaging within each fraction, whereas multi-IMPT distributes angular sampling across fractions. Although these temporal patterns differ, the cumulative BED formulation biologically couples fractions by explicitly summing per-fraction dose contributions and penalizing larger per-fraction doses through its quadratic term. As shown in the per-fraction analysis, multi-IMPT did not produce excessive single-fraction OAR dose concentration under the evaluated conditions. Consequently, additional temporal smoothing constraints were not required for the studied cases. Nevertheless, in hypofractionated settings, scenarios with higher per-fraction doses, or cases with tightly binding OAR constraints, temporal dose heterogeneity may play a more pronounced biological role. The per-fraction analysis for DVH-derived metrics presented in this study is based on a cyclic delivery of a finite number of beam-angle subsets, which provides a structured approximation of temporal angular averaging. However, this periodic delivery pattern is an idealization, and in clinical settings, fraction-to-fraction variations in anatomy and setup may disrupt this regularity. Such variations could lead to differences in voxel-wise temporal dose accumulation that are not captured in the present analysis. Furthermore, the observed agreement between multi-IMPT and ARC in terms of cumulative BED for the limited number of cases should be interpreted as an empirical finding under the evaluated cases and planning conditions, rather than as a property that is theoretically guaranteed by the formulation. In such contexts, incorporation of explicit per-fraction constraints or temporal regularization terms followed by voxel-wise dose tracking across fractions may provide additional insights and represents an important direction for future investigation.

The relative performance of multi-IMPT appears to depend on anatomical and geometric characteristics of the treatment site. In general, fraction-varying beam strategies are expected to provide greater benefit in cases where critical OAR are distributed circumferentially around the target and sparing relies on distributing entrance dose across multiple beam directions rather than avoiding a single dominant orientation. This behavior is consistent with the prostate case, where multi-IMPT achieved substantial reductions in OAR BED. In contrast, for anatomically constrained sites such as the brain and head-and-neck, where conformity is strongly influenced by specific beam orientations, the relative advantage of angular redistribution may be reduced. A systematic investigation of predictive anatomical or dosimetric factors, such as angular OAR overlap metrics, geometric proximity indices, or conformity demands, would require analysis of a larger patient cohort and remains an important direction for future work.

The beam angle subset selection used in this study represents 1 clinically reasonable instantiation of the proposed multi-IMPT framework rather than a uniquely optimal configuration. The choice of 4 beams per fraction and interlaced 15° angular offsets was motivated by a balance between approximating the angular averaging effect of proton ARC, maintaining clinically practical per-fraction delivery complexity, and ensuring computational feasibility. While the dose distribution produced by each IMPT plan depends on the selected beam angles, the proposed framework itself does not rely on a specific number of beams per fraction or a particular angular spacing. Alternative configurations using different beam counts or angular offsets can be accommodated within the same optimization formulation. A systematic sensitivity analysis evaluating the impact of beam angle subset size and angular spacing on BED distributions and robustness is beyond the scope of this study and will be investigated in the future work.

Additionally, the beam arrangements used in this study were selected to ensure consistent angular sampling across IMPT, ARC, and multi-IMPT, rather than to reflect clinically optimized beam selection. Uniformly distributed beam angles for all methods enable an unbiased comparison but may include beam paths not typically preferred in clinical practice. However, all methods were optimized using identical objectives, constraints and the same BED-based formulation, ensuring that differences arise from the delivery strategy rather than beam selection. Therefore, the relative comparison remains meaningful and internally consistent, although incorporation of optimized beam arrangements represents an important step towards clinical implementation of the proposed method.

From a practical implementation perspective, multi-IMPT differs from conventional single-plan IMPT workflows because current commercial proton treatment planning systems do not natively support fraction-dependent spot intensities within a single plan. In practice, the proposed approach would be implemented as multiple pre-approved fixed-beam IMPT plans delivered in a predefined cyclic sequence. Each subplan is a standard deliverable IMPT plan and can be executed using existing scanning hardware without modification. Because 6 subplans are used in this study, 6 corresponding patient-specific QA measurements would be required prior to treatment initiation. These QA procedures can be performed in batch before the first fraction, similar to workflows used for adaptive radiotherapy or sequential boost treatments. Modern record-and-verify systems can automate fraction-specific plan scheduling, thereby reducing the risk of delivery errors during plan switching.

Although multi-IMPT increases upfront planning and QA workload relative to a single-plan IMPT approach, it does not increase per-fraction delivery time or scanning complexity. Per-fraction spot counts and energy layers remain comparable to standard IMPT and substantially lower than ARC, which activates a large number of beam angles within each fraction. Thus, multi-IMPT shifts complexity toward pre-treatment preparation rather than on-table delivery. It is also acknowledged that proton ARC may offer additional advantages related to intra-fraction angular averaging and potential LET modulation, which were not explicitly modeled in this study. Evaluation of LET-weighted biological effects within a fraction-varying framework remains an important direction for future work.

Deliverability analysis in this study was performed using plan complexity metrics, including per-fraction spot count, total spot weights, and number of energy layers. These metrics provide a practical proxy for delivery burden and energy-switching complexity. The results show that multi-IMPT maintains per-fraction spot counts and energy layer numbers comparable to standard IMPT, whereas ARC exhibits substantially higher per-fraction complexity due to the simultaneous activation of all beam angles. Although multi-IMPT requires longer optimization time than single-plan IMPT (approximately 6 times longer due to the use of 6 distinct IMPT plans), its computational burden remains consistently lower than that of ARC. Thus, multi-IMPT preserves IMPT-like per-fraction delivery characteristics while approximating the cumulative angular sampling of ARC under the evaluated conditions. However, it is important to note that deliverability was assessed using plan complexity metrics only. Machine-level delivery validation, treatment log-file analysis, energy-switch timing evaluation, interplay assessment, and experimental patient-specific QA measurements were not performed in this study. A comprehensive clinical implementation assessment would require hardware-specific delivery testing and workflow validation, which represents an important direction for future work.

A robustness analysis incorporating ±3 mm setup and ±3.5% range uncertainties further demonstrates that multi-IMPT exhibits stability comparable to ARC and, in several scenarios, improved target dose homogeneity relative to standard IMPT. Variability in OAR BED metrics remained modest across uncertainty scenarios, indicating that fraction-varying beam delivery does not introduce increased sensitivity under clinically realistic perturbations. However, the current analysis remains limited since uncertainty is modeled to be static and applied independently across scenarios. In particular, the present framework evaluates perturbed dose distributions on a per-scenario basis without explicitly accounting for fraction-to-fraction variation or temporal accumulation of uncertainties. This limitation is especially relevant for multi-IMPT, where different beam-angle subsets are delivered across fractions and uncertainty effects may accumulate in a fraction-dependent manner. As a result, the current analysis does not capture potential temporal interplay between delivery variations and uncertainty. Incorporating fraction-dependent uncertainty modeling, including stochastic or voxel-wise accumulation of dose across fractions, may provide a more comprehensive characterization of robustness and represents an important direction for future investigation.

Overall, the primary contribution of this work lies in the development of a BED-optimized, fraction-varying beam delivery framework that approximates ARC-level biological performance while preserving IMPT-like per-fraction complexity. The results suggest that multi-IMPT represents a clinically feasible alternative in settings where proton ARC delivery is not available, with performance that is robust, case-consistent within clinical tolerance limits, and adaptable to different beam configurations.

## Conclusions

This work presents a BED-optimized multi-IMPT framework that distributes beam-angle subsets across fractions to approximate the cumulative angular coverage of proton ARC while preserving fixed-beam IMPT deliverability. Across 4 clinical cases, multi-IMPT achieved cumulative BED and target coverage comparable to ARC under conventional fractionation, with modest OAR sparing improvements in selected geometries. Per-fraction analysis confirmed that multi-IMPT did not produce excessive single-fraction OAR dose concentration, and robustness evaluation under setup and range uncertainties demonstrated stability comparable to ARC. Deliverability assessment using plan complexity metrics showed IMPT-like per-fraction spot counts and energy layers, substantially lower than ARC. Under the evaluated conditions, multi-IMPT provides ARC-comparable cumulative biological performance and represents a viable alternative to ARC.

## Ethics

This research was carried out under Human Subject Assurance Number 00003411 for the University of Kansas in accordance with the principles embodied in the Declaration of Helsinki and in accordance with local statutory requirements.

## Funding

This research is partially supported by the NIH grants No. R37CA250921, R01CA261964.

## CRediT authorship contribution statement

Nimita Shinde: Formal analysis, Investigation, Methodology, Validation, Visualization, Writing – original draft. Yanan Zhu: Methodology, Validation, Writing – review and editing. Wei Wang: Methodology, Validation, Writing – review and editing. Wangyao Li: Data curation, Resources, Writing – review and editing. Yuting Lin: Data curation, Resources, Validation, Writing – review and editing. Gregory N. Gan: Data curation, Resources, Validation, Writing – review and editing. Christopher Lominska: Data curation, Resources, Writing – review and editing. Ronny Rotondo: Data curation, Resources, Writing – review and editing. Ronald C. Chen: Resources, Writing – review and editing. Hao Gao: Conceptualization, Funding acquisition, Investigation, Methodology, Project administration, Supervision, Writing – review and editing.

## Declaration of Generative AI and AI-Assisted Technologies in the Writing Process

During the preparation of this work, the authors used chatGPT in order to polish certain parts of the manuscript. After using this tool/service, the authors reviewed and edited the content as needed and take full responsibility for the content of the published article.

## Declaration of Competing Interest

The authors declare that they have no known competing financial interests or personal relationships that could have appeared to influence the work reported in this paper.

## Data Availability

The authors are not able to share the data at this time.

## Appendix A: ICR and ADMM method (Algorithm 1) for solving the augmented Lagrangian formulation (Equation (3))

In this section, the steps of Algorithm 1 are explained in detail. In Step 1, the values of the parameters in the augmented Lagrangian model are carefully chosen. The weights associated with the least-squares objective terms ($w_{i}$’s) were selected via grid search from the set {0.01, 0.1, 1, 10, 100}, and the augmented Lagrangian penalty coefficients ($\mu_{i}$*’s*) were selected via grid search from the set: {0.01, 10^−3^, 10^−4^, 10^−5^}. In Step 2, the decision variable $u_{t}$ is randomly initialized. Specifically, in the experiments in this work, $u_{t}$ is set to $u_{t}=0\;\forall t$. In Step 3, remaining primal and dual variables are initialized.

Next, in each iteration k, Step 4a defines the active index set of the BED-DVH max and DVH min constraints as explained in Section 2.1. The BED-DVH and DVH-min constraints are defined for OAR voxels and target voxels, respectively, based on the active index set. Finally, Step 4c updates the dual variables in the augmented Lagrangian formulation (Equation (3)) and Step 4b defines the updates to the primal variables. The procedure to update each primal variable is given below.

1. Updating $u_{t}$: For each $t\in \left[T\right],$ fix all variables except $u_{t}$. The augmented Lagrangian formulation (Equation (3)) is then unconstrained in $u_{t}$. Thus, to solve the problem, take the first-order derivative of the objective function in Equation (3) and set it to 0. Then, $u_{t}$ is the solution of the resulting linear system of equations.
2. Updating $y_{t}$: For each $t\in \left[T\right],$ fix all variables except $y_{t}$ in Equation (3). The resulting optimization problem has a closed-form solution as follows: $y_{t}=\max\left(g,u_{t}+\zeta_{t}\right)$ if $u_{t}+\zeta_{t}\geq g/2$. Otherwise, $y_{t}=0$.
3. Updating $z^{\mathrm{tm}}$ for all $m\in M_{1}$: For each $m\in M_{1}$, $j\in \left[n_{m}\right]$, fix all variables except $z_{j}^{\mathrm{tm}}$ in Equation (3). The resulting minimization problem over $z_{j}^{\mathrm{tm}}$ is
  $$\begin{array}{c} \min\sum_{t=1}^{T}{\left(A_{j}^{\mathrm{tm}}u_{t}+\lambda_{j}^{\mathrm{tm}}-z_{j}^{\mathrm{tm}}\right)}^{2}\;s.t.\sum_{t=1}^{T}{\left(z_{j}^{\mathrm{tm}}+\frac{1}{2\rho_{m}}\right)}^{2}\leq \frac{\mathrm{BE}D_{\max}^{m}}{\rho_{m}}+\frac{T}{4\rho_{m}^{2}}. \end{array}$$
  The optimal solution to the resulting minimization problem is the projection of $A_{j}^{\mathrm{tm}}u_{t}+\lambda_{j}^{\mathrm{tm}}$ onto the quadratic inequality constraint. The projected point (ie, the optimal solution) is defined as $z_{j}^{\mathrm{tm}}=\left(1-r\right)\left(A_{j}^{\mathrm{tm}}u_{t}+\lambda_{j}^{\mathrm{tm}}\right)-r\frac{1}{2\rho_{m}}$, where r=max{0,q} and q can be defined as follows: $q=1-\sqrt{\frac{\frac{\mathrm{BE}D_{\max}^{m}}{\rho_{m}}-+\frac{T}{4\rho_{m}^{2}}}{\sum_{t=1}^{T}{\left(A_{j}^{\mathrm{tm}}u_{t}+\lambda_{j}^{\mathrm{tm}}+\frac{1}{2\rho_{m}}\right)}^{2}}}$.
4. Updating $z^{\mathrm{tm}}$ for all $m\in M_{2}$: For each $m\in M_{2}$, fix all variables except $z_{j}^{\mathrm{tm}}$ in Equation (3). The resulting minimization problem over $z_{j}^{\mathrm{tm}}$ is
  $$\begin{array}{c} \min\sum_{t=1}^{T}\sum_{j=1}^{n_{m}}{\left(A_{j}^{\mathrm{tm}}u_{t}+\lambda_{j}^{\mathrm{tm}}-z_{j}^{\mathrm{tm}}\right)}^{2}\;s.t.\sum_{t=1}^{T}\sum_{j=1}^{n_{m}}{\left(z_{j}^{\mathrm{tm}}+\frac{1}{2\rho_{m}}\right)}^{2}\leq \frac{n_{m}\mathrm{BE}D_{\mathrm{mean}}^{m}}{\rho_{m}}+\frac{Tn_{m}}{4\rho_{m}^{2}}. \end{array}$$
  The optimal solution to this problem is the projection of $A_{j}^{\mathrm{tm}}u_{t}+\lambda_{j}^{\mathrm{tm}}$ onto the inequality constraint. Thus, the optimal solution is $z_{j}^{\mathrm{tm}}=\left(1-r\right)\left(A_{j}^{\mathrm{tm}}u_{t}+\lambda_{j}^{\mathrm{tm}}\right)-r\frac{1}{2\rho_{m}}$, where r=max{0,q} and $q=1-\sqrt{\frac{\frac{n_{m}\mathrm{BE}D_{\mathrm{mean}}^{m}}{\rho_{m}}-+\frac{Tn_{m}}{4\rho_{m}^{2}}}{\sum_{t=1}^{T}\sum_{j=1}^{n_{m}}{\left(A_{j}^{\mathrm{tm}}u_{t}+\lambda_{j}^{\mathrm{tm}}+\frac{1}{2\rho_{m}}\right)}^{2}}}$.
5. Updating $z^{\mathrm{tm}}$ for all $m\in M_{3}$: For each $m\in M_{3}$, $j\in \Omega^{m}$, $z^{\mathrm{tm}}$ can be updated using the same procedure as outlined for updating $z^{\mathrm{tm}}$ for $m\in M_{1}$.
6. Updating $z^{t0}$: For each $j\in \left[n_{0}\right],$ fix all variables except $z_{j}^{t0}$ in Equation (3). The resulting minimization problem is

$$\begin{array}{c} \min\sum_{t=1}^{T}{\left(A_{j}^{t0}u_{t}+\lambda_{j}^{t0}-z_{j}^{t0}\right)}^{2}\;s.t.\sum_{t=1}^{T}{\left(z_{j}^{t0}+\frac{1}{2\rho_{0}}\right)}^{2}\geq \frac{\mathrm{BE}D_{\mathrm{dv}}^{0}}{\rho_{0}}+\frac{T}{4\rho_{0}^{2}}. \end{array}$$

The optimal solution to this problem is the projection of $A_{j}^{t0}u_{t}+\lambda_{j}^{t0}$ onto the constraint. Thus, the optimal solution to the above problem is $z_{j}^{t0}=\left(1+r\right)\left(A_{j}^{t0}u_{t}+\lambda_{j}^{t0}\right)+r\frac{1}{2\rho_{0}}$, where r=max{0,q} and $q=\sqrt{\frac{\frac{\mathrm{BE}D_{\mathrm{dv}}^{0}}{\rho_{0}}-+\frac{T}{4\rho_{0}^{2}}}{\sum_{t=1}^{T}{\left(A_{j}^{t0}u_{t}+\lambda_{j}^{t0}+\frac{1}{2\rho_{0}}\right)}^{2}}}-1$.

## Footnotes

Appendix A Supplementary data associated with this article can be found in the online version at doi:10.1016/j.ijpt.2026.101321.
