# Supplementary material for: Biologically Effective Dose-Optimized Multi-Intensity-Modulated Proton Therapy: A Biologically Comparable Alternative to Proton Arc Therapy
Source: Int J Part Ther. 2026 May 8;20:101321. doi: 10.1016/j.ijpt.2026.101321 (PMC13208107; doi:10.1016/j.ijpt.2026.101321)
Supplement: Supplementary file 1 — Supplementary material [file mmc1.docx]

**Supplementary material: BED-optimized multi-IMPT: a biologically comparable alternative to proton ARC therapy**

**Section A: Sensitivity of Optimization to** $\boldsymbol{\alpha/\beta}$ **ratio**

**Experimental Setup:** To evaluate the sensitivity of the proposed BED-based optimization framework to the assumed radiobiological parameters, additional experiments were performed using $\alpha/\beta$ values of 1 Gy and 4 Gy, in addition to the nominal value of 2 Gy used in the primary analysis. For each clinical case and for each planning technique (IMPT, ARC, and multi-IMPT), the optimization was repeated independently using the alternative $\alpha/\beta$ values.

Clinical OAR constraints were defined in terms of physical dose and were kept unchanged. For each $\alpha/\beta$ value, the corresponding BED upper bounds were recalculated using the linear-quadratic model (given in Section 2 of the main manuscript), and the optimization problem was re-solved with identical solver settings and normalization criteria. All plans were normalized such that at least 95% of the target received 100% of the prescribed dose.

**Results:** Across all cases and techniques, the optimized physical dose distributions were invariant with respect to the selected $\alpha/\beta$ value. Consequently, target Dmax (reported as percentage of prescription) remained unchanged. Differences observed in OAR BED values reflect the expected rescaling within the BED formula rather than changes in the underlying optimized physical dose. Importantly, the relative ranking and comparative differences between IMPT, ARC, and multi-IMPT remained consistent across $\alpha/\beta$ values. This limited sensitivity reflects the clinical context of the evaluated cases. All treatments were conventionally fractionated (1.2-2 Gy per fraction), and OAR constraints were not tightly active at their upper bounds. Under these conditions, the quadratic component of the BED formulation contributes modestly relative to the linear dose term, and variation in $\alpha/\beta$ primarily rescales BED values without altering the physical dose trade-offs determined by the optimization.

Fractionation effects are expected to play a more pronounced role in scenarios involving hypofractionation, higher per-fraction doses, very low $\alpha/\beta$ tissues, or tightly binding OAR constraints. In such settings, the quadratic BED term and temporal dose heterogeneity may significantly influence optimization outcomes. The present analysis therefore indicates that, for conventionally fractionated regimens with non-binding OAR constraints, $\alpha/\beta$ sensitivity is limited, while the proposed BED-based framework remains applicable and potentially more impactful in biologically driven scenarios. Tables S1-S4 summarize the resulting BED metrics across $\alpha/\beta$ values for all techniques and cases.

Table S1 (Prostate): Sensitivity of the optimization methods to $\alpha/\beta$. OAR BED values are reported in Gy.

| **Structure** | **Quantity** | **IMPT** | | | **ARC** | | | **multi-IMPT** | | |
| --- | --- | --- | --- | --- | --- | --- | --- | --- | --- | --- |
|  |  | $\alpha/\beta=1$Gy | $\alpha/\beta=2$ Gy | $\alpha/\beta=4$ Gy | $\alpha/\beta=1$ Gy | $\alpha/\beta=2$ Gy | $\alpha/\beta=4$ Gy | $\alpha/\beta=1$ Gy | $\alpha/\beta=2$ Gy | $\alpha/\beta=4$ Gy |
| CTV | CI | 0.76 | 0.76 | 0.76 | 0.76 | 0.76 | 0.76 | 0.78 | 0.78 | 0.78 |
|  | D_max_ (%) | 113.62 | 113.62 | 113.62 | 110.74 | 110.74 | 110.74 | 106.60 | 106.60 | 106.60 |
| Bladder | BED_50_ | 25.87 | 21.27 | 18.97 | 59.34 | 44.60 | 37.23 | 30.52 | 24.49 | 21.51 |
|  | BED_20_ | 72.63 | 53.38 | 43.75 | 94.95 | 67.75 | 54.15 | 80.91 | 58.65 | 47.47 |
| Rectum | BED_50_ | 29.77 | 24.12 | 21.30 | 59.97 | 45.02 | 37.55 | 30.68 | 24.69 | 21.65 |
|  | BED_20_ | 73.18 | 53.74 | 44.01 | 91.72 | 65.70 | 52.69 | 71.24 | 52.46 | 43.06 |
|  | BED_10_ | 109.74 | 77.09 | 60.80 | 116.77 | 81.48 | 63.84 | 103.87 | 73.39 | 58.15 |
| Fem head | BED_10_ | 13.27 | 11.62 | 10.80 | 2.84 | 2.73 | 2.67 | 4.67 | 4.14 | 3.87 |
| Penile bulb | BED_50_ | 12.18 | 10.57 | 10.03 | 14.81 | 12.85 | 11.87 | 13.13 | 11.51 | 10.70 |

Table S2 (Lung): Sensitivity of the optimization methods to $\alpha/\beta$. OAR BED values are reported in Gy.

| **Structure** | **Quantity** | **IMPT** | | | **ARC** | | | **multi-IMPT** | | |
| --- | --- | --- | --- | --- | --- | --- | --- | --- | --- | --- |
|  |  | $\alpha/\beta=1$Gy | $\alpha/\beta=2$ Gy | $\alpha/\beta=4$ Gy | $\alpha/\beta=1$ Gy | $\alpha/\beta=2$ Gy | $\alpha/\beta=4$ Gy | $\alpha/\beta=1$ Gy | $\alpha/\beta=2$ Gy | $\alpha/\beta=4$ Gy |
| CTV | CI | 0.867 | 0.867 | 0.867 | 0.937 | 0.937 | 0.937 | 0.929 | 0.929 | 0.929 |
|  | D_max_ (%) | 128.02 | 128.02 | 128.02 | 113.39 | 113.39 | 113.39 | 109.31 | 109.31 | 109.31 |
| Lung | BED_mean_ | 9.18 | 7.10 | 6.05 | 7.93 | 6.24 | 5.39 | 9.11 | 6.97 | 5.91 |
|  | BED_30_ | 3.08 | 3.07 | 3.07 | 3.36 | 3.23 | 3.16 | 3.78 | 3.43 | 3.26 |
| Heart | BED_mean_ | 2.26 | 1.76 | 1.51 | 2.91 | 2.28 | 1.97 | 3.29 | 2.54 | 2.16 |
| Esophagus | BED_mean_ | 5.82 | 5.08 | 4.71 | 5.72 | 5.06 | 4.73 | 6.06 | 5.29 | 4.91 |

Table S3 (Brain): Sensitivity of the optimization methods to $\alpha/\beta$. OAR BED values are reported in Gy.

| **Structure** | **Quantity** | **IMPT** | | | **ARC** | | | **multi-IMPT** | | |
| --- | --- | --- | --- | --- | --- | --- | --- | --- | --- | --- |
|  |  | $\alpha/\beta=1$Gy | $\alpha/\beta=2$ Gy | $\alpha/\beta=4$ Gy | $\alpha/\beta=1$ Gy | $\alpha/\beta=2$ Gy | $\alpha/\beta=4$ Gy | $\alpha/\beta=1$ Gy | $\alpha/\beta=2$ Gy | $\alpha/\beta=4$ Gy |
| CTV | CI | 0.838 | 0.838 | 0.838 | 0.904 | 0.904 | 0.904 | 0.863 | 0.863 | 0.863 |
|  | D_max_ (%) | 111.76 | 111.76 | 111.76 | 103.14 | 103.14 | 103.14 | 104.08 | 104.08 | 104.08 |
| Brainstem | BED_max_ | 84.81 | 72.41 | 66.21 | 85.25 | 72.75 | 66.50 | 87.91 | 74.75 | 68.17 |
|  | BED_mean_ | 14.37 | 13.03 | 12.36 | 14.95 | 13.52 | 12.80 | 16.88 | 15.11 | 14.23 |
| Brain | BED_mean_ | 113.28 | 94.03 | 84.40 | 104.31 | 87.30 | 78.79 | 109.37 | 91.10 | 81.96 |
|  | BED_mean_ | 1.05 | 0.97 | 0.92 | 1.01 | 0.93 | 0.88 | 1.11 | 1.01 | 0.96 |

Table S4 (HN): Sensitivity of the optimization methods to $\alpha/\beta$. OAR BED values are reported in Gy.

| **Structure** | **Quantity** | **IMPT** | | | **ARC** | | | **multi-IMPT** | | |
| --- | --- | --- | --- | --- | --- | --- | --- | --- | --- | --- |
|  |  | $\alpha/\beta=1$Gy | $\alpha/\beta=2$ Gy | $\alpha/\beta=4$ Gy | $\alpha/\beta=1$ Gy | $\alpha/\beta=2$ Gy | $\alpha/\beta=4$ Gy | $\alpha/\beta=1$ Gy | $\alpha/\beta=2$ Gy | $\alpha/\beta=4$ Gy |
| CTV | CI | 0.74 | 0.74 | 0.74 | 0.85 | 0.85 | 0.85 | 0.79 | 0.79 | 0.79 |
|  | D_max_ (%) | 107.40 | 107.40 | 107.40 | 102.19 | 102.19 | 102.19 | 102.45 | 102.45 | 102.45 |
| R Parotid | BED_max_ | 2.42 | 2.36 | 2.33 | 5.95 | 5.62 | 5.45 | 6.35 | 5.85 | 5.61 |
| Oral Cavity | BED_max_ | 216.10 | 146.45 | 111.62 | 206.33 | 140.5 | 107.58 | 205.15 | 140.21 | 106.74 |
|  | BED_mean_ | 15.60 | 12.38 | 10.77 | 13.32 | 10.95 | 9.75 | 15.13 | 12.14 | 10.64 |
| Oropharynx | BED_mean_ | 78.60 | 56.71 | 46.77 | 77.55 | 55.97 | 45.17 | 84.17 | 60.19 | 48.20 |
| Larynx | BED_mean_ | 6.15 | 4.50 | 3.68 | 6.48 | 4.73 | 3.85 | 7.09 | 5.13 | 4.15 |

**Section B: Robustness analysis under setup and range uncertainties**

This section complements Section 3.3 of the main manuscript by providing full robustness results for the brain and head-and-neck cases. Mean ± standard deviation values for all evaluated dosimetric metrics are reported, along with corresponding BED-DVH plots under uncertainty (Figure S1). In addition, worst-case values are reported for all evaluated metrics to provide a more clinically relevant robustness assessment. These results are consistent with the trends described in the main manuscript and confirm comparable robustness between ARC and multi-IMPT across all clinical scenarios evaluated.

**Section C: Per-fraction physical dose analysis**

This section complements Section 3.4 of the main manuscript by providing per-fraction physical dose metrics for the brain and HN cases (Table S5). As in the main analysis, IMPT and ARC deliver identical dose distributions each fraction, whereas multi-IMPT statistics are computed across the six cyclically delivered subplans. For the brain case, multi-IMPT demonstrated modest inter-subplan variability without evidence of excessive single-fraction OAR dose concentration. Brainstem maximum dose across subplans was 1.00 ± 0.05 Gy (maximum 1.05 Gy), compared to 0.91 Gy for ARC. Brain mean dose values showed negligible variation across the six subplans. Target maximum dose variability remained controlled and comparable to ARC.

In the HN case, similar trends were observed. Oral cavity Dmax was 2.00 ± 0.04 Gy (maximum 2.06 Gy), and oropharynx Dmean was 0.94 ± 0.06 Gy for multi-IMPT. These values were comparable to ARC and did not indicate excessive single-fraction dose spikes. Overall, the brain and HN results are consistent with the findings reported in Section 3.4 and further support that multi-IMPT does not introduce excessive temporal dose heterogeneity under conventional fractionation.


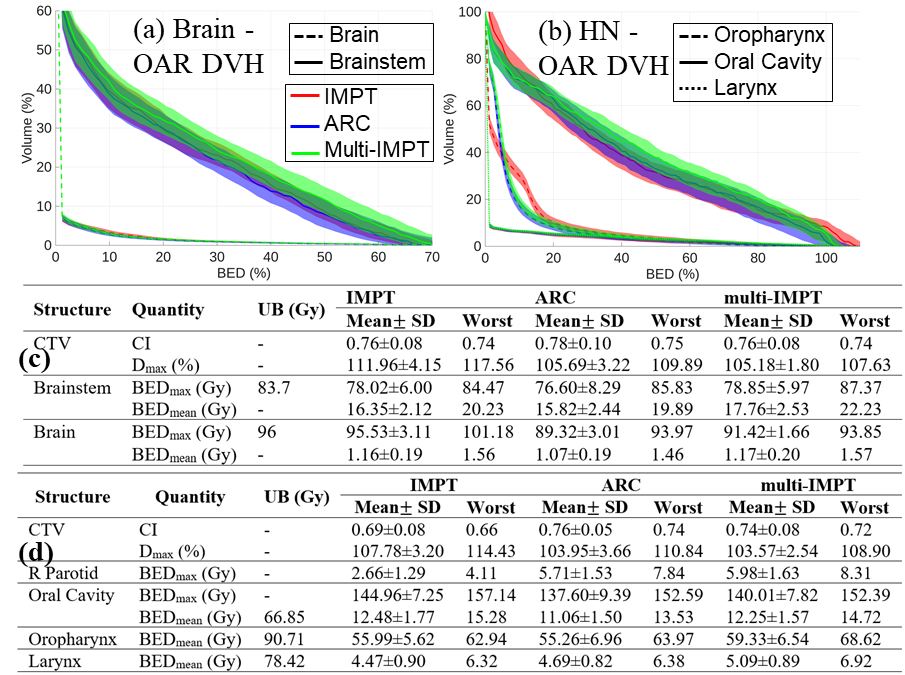


Figure S1. Uncertainty analysis for the brain and HN cases. (a)-(b) OAR BED-DVH for brain and HN, respectively. (c)-(d) Dosimetric comparison showing mean $\pm$ 1 standard deviation and worst-case values across uncertainty scenarios for brain and HN, respectively.

Table S5: Per-fraction physical dose metrics for brain and HN cases. Since IMPT and ARC deliver identical dose each fraction, a single per-fraction value is shown. Multi-IMPT statistics are computed across the six cyclically delivered subplans, with both the maximum single-fraction value and mean ± SD reported to quantify temporal dose variation. All OAR physical dose values are reported in Gy.

| **Case** | **Structure** | **Quantity** | **IMPT (per-fraction)** | **ARC (per-fraction)** | **multi-IMPT (max across six subplans)** | **multi-IMPT (mean ± SD across six subplans)** |
| --- | --- | --- | --- | --- | --- | --- |
| Brain | CTV | D_max_ (%) | 111.76 | 103.14 | 110.66 | 106.44±2.17 |
|  | Brainstem | D_max_ | 0.91 | 0.91 | 1.05 | 1.00±0.05 |
|  |  | D_mean_ | 0.17 | 0.18 | 0.22 | 0.20±0.02 |
|  | Brain | D_max_ | 1.13 | 1.06 | 1.19 | 1.14±0.02 |
|  |  | D_mean_ | 0.01 | 0.01 | 0.01 | 0.01±0.001 |
| HN | CTV | D_max_ (%) | 107.40 | 102.19 | 106.92 | 104.10±1.43 |
|  | R Parotid | D_max_ | 0.05 | 0.13 | 0.21 | 0.17±0.09 |
|  | Oral Cavity | D_max_ | 1.99 | 1.93 | 2.06 | 2.00±0.04 |
|  |  | D_mean_ | 0.23 | 0.22 | 0.30 | 0.23±0.04 |
|  | Oro-pharynx | D_mean_ | 0.86 | 0.89 | 1.02 | 0.94±0.06 |
|  | Larynx | D_mean_ | 0.07 | 0.07 | 0.09 | 0.08±0.01 |
